# Purification, characterization and *in vitro* and *in vivo* immune enhancement of polysaccharides from mulberry leaves

**DOI:** 10.1371/journal.pone.0208611

**Published:** 2019-01-02

**Authors:** Xiaolan Chen, Zhicun Sheng, Shulei Qiu, Haifeng Yang, Jiping Jia, Jing Wang, Chunmao Jiang

**Affiliations:** Jiangsu Agri-animal Husbandry Vocational College, Taizhou, Jiangsu Province, China; National Institute of Animal Biotechnology, INDIA

## Abstract

Mulberry leaf polysaccharide (MLP) was extracted and purified by DEAE-52 cellulose and Sephadex G-100 column chromatography to afford two major purified polysaccharides (MLP-1 and MLP-2). The purified polysaccharides were characterized, and their immune-enhancing properties were investigated. MLP-1 had a molecular weight of 9.31×10^4^ Da and was composed of mannose, rhamnose, glucose, galactose, xylose, and arabinose in a molar ratio of 0.71:1.00:2.76:1.13:3.70:2.81. The molecular weight of MLP-2 was 2.22×10^6^ Da, and its monosaccharide constituents were mannose, rhamnose, glucose, galactose, and arabinose in a molar ratio of 1.31:8.45:6.94:1.00:11.96. Infrared spectroscopy showed that each MLP had a typical absorption peak characteristic of sugars, and ultraviolet (UV) spectroscopy showed that neither MLP contained nucleic acid or protein components. Then, the abilities of these polysaccharides to stimulate spleen lymphocyte proliferation in mice *in vitro* were compared by the 3-[4,5-dimethylthiazol-2-yl]-2,5-diphenyltetrazolium bromide (MTT) assay. MLP-2 was more effective than MLP-1; therefore, MLP-2 was chosen for the study of its immune-enhancing effects *in vivo*. For the *in vivo* experiments, 14-day-old chickens immunized with Newcastle disease (ND) vaccine were orally administered MLP-2, and *Astragalus* polysaccharide (APS) was used as the control. Each chicken was orally administered 4 mg or 8 mg of MLP-2 for seven consecutive days starting three days before ND vaccine immunization. MLP-2 significantly improved the ND serum antibody titer and interleukin-2 (IL-2), interferon-γ (IFN-γ) and immunoglobulin A (sIgA) concentrations in tracheal and jejunal wash fluids, and increasing numbers of immune globulin A-positive (IgA^+^) cells in cecal tonsils and increased body weight. These results indicated that MLP-2 could significantly enhance immune activity and could therefore be utilized as an immunopotentiator drug candidate.

## Introduction

The modulation of the immune response plays an important role in preventing diseases, and increasing research attention has been paid to the immunomodulation and immunostimulation induced by active substances [1]. Current immunomodulators include immune adjuvants such as aluminum hydroxide, Freund's adjuvant (FA) and albumen adjuvants. However, neither aluminum hydroxide nor FA can induce strong cellular immunity, and FIA can cause local stimulation, tissue damage, and even carcinogenesis [2]. Albumen adjuvants were developed recently but were too expensive to be commercialized. Therefore, there is an urgent need to research and develop a new-type immune adjuvant with high efficiency, low toxicity and extensive resources [3].

Many Chinese herbal polysaccharides have obvious advantages in improving humoral immunity and cellular immunity, such as *Cyrtomium macrophyllum* [4], *Cassia obtusifolia* [5], *Atractylodes macrocephala* Koidz (RAMPS) [6], RAMPStp and RAMPS60c [7]. Numerous Chinese herbal polysaccharides can also enhance the mucosal immunity of animals. For example, oral administration of Si Jun Zi Tang polysaccharide can increase the number of IgA ^+^ cells in the small intestine in mice and enhance mucosal immunity [8]. Therefore, Chinese herbal polysaccharides have obvious benefits in improving humoral immunity, cellular immunity and mucosal immunity and could become promising compounds in the development of immunomodulators.

Mulberry (*Morus alba*) can grow very well under various climatic conditions ranging from temperate to tropical. Mulberry leaves can be used to feed silkworms, and it contains many active ingredients with a wide range of pharmacological activities. For example, rutin and quercetin in mulberry leaves can enhance antioxidative activity and treat hyperglycemia [9]. Deoxynojirimycin has α-glucosidase inhibitory activity, allowing it to reduce blood glucose levels [10]. Most of these active ingredients are found in the ethanol extracts of mulberry leaves. Mulberry leaf polysaccharide (MLP), another major active component, can promote insulin expression and modulate hepatic glucose metabolism in alloxan-induced diabetic mice [11]. To some extent, MLP can also act as an antioxidant and antibacterial agent [12]. In veterinary clinics, adding MLP to pig feed during early weaning can improve intestinal microbial ecology, reduce the occurrence of diarrhea, and improve overall growth [13].

In addition to the above effects, MLP can significantly improve the carbon clearance capacity and spleen lymphocyte transformation capability induced by ConA and can notably increase the thymus index [14]. Furthermore, previous studies have shown that the porcine reproductive and respiratory syndrome (PRRS)-specific antibody titer could be significantly improved when pigs immunized with a PRRS vaccine were orally administered MLP [15], which indicates that MLP has some immunomodulatory properties.

In this study, MLP was extracted by the ethanol precipitation method and further purified by DEAE-cellulose-52 and Sephadex G-100 column chromatography to afford two major purified polysaccharides (MLP-1 and MLP-2). The purified polysaccharides were characterized, and their immune properties were investigated. The purpose of this study was to investigate the effects of purified MLP-1 and MLP-2 on immune cells *in vitro* and on humoral immunity and respiratory and intestinal mucosal immunity in chickens *in vivo*. This study provides a theoretical basis for the development of new animal immunopotentiators.

## Materials and methods

### Drug preparation

The mulberry leaves were purchased from a mulberry plantation in Bozhou (Anhui, China), washed with tap water, and air dried at room temperature. MLP was extracted as follows: temperature of 100°C, time of 2 hours, and ratio of water to raw material of 25 mL/g. The resulting solution was filtered, and the supernatant was mixed with ethanol in a 1:3 (v/v) ratio and stored overnight. The precipitates were collected by centrifugation at 8000 rpm for 15 min, washed with absolute ethanol and lyophilized to afford the crude MLP, which was further purified.

For the *in vitro* studies, MLP-1 and MLP-2 were diluted with RPMI-1640 containing 10% fetal bovine serum to five working concentrations (250–15.625 μg/mL), sterilized and stored at 4°C. For the *in vivo* studies, based on the results of the *in vitro* studies, a 2 mg/mL solution of MLP-2 was prepared in distilled water, sterilized and stored at 4°C. APS, Lot No. 20150124, was produced by Beijing Health Life Technology Co., Ltd.

### Reagents and vaccine

1-Phenyl-3-methyl-4-benzene formyl pyrazolone (PMP) was purchased from Sigma-Aldrich (St. Louis, MO, USA). The reference monosaccharides (mannose, glucose, D-ribose, rhamnose, D-xylose, D-galactose, L-arabinose, and D-fructose) and standard dextrans (T10, T40, T70, T380, and T500) were all purchased from Solarbio Co., Ltd. (Beijing, China). Hanks’ solution from Wuhan Biohao Biotechnology Co., Ltd. was used to dilute the blood. RPMI-1640 (GIBCO) was used for culturing cells. Fetal bovine serum (Australia, No. 10099254) was added to RPMI-1640 as a source of nutrition for cell growth. MTT (Sigma, USA) was dissolved in PBS (5 mg/mL, pH 7.4). Dimethyl sulfoxide (DMSO, No. 20150619) was acquired from Sinopharm Chemical Reagent Co., Ltd. APS, Lot No. 20150124, was purchased from Beijing Health Life Technology Co., Ltd. Phytohemagglutinin (PHA, Sigma Company, No. L-8653), a T-cell mitogen, was diluted to 0.1 mg/mL with RPMI-1640. Lipopolysaccharide (LPS) (Sigma, No. L3224), a B-cell mitogen, was diluted to 0.1 mg/mL with RPMI-1640. Red blood cell lysis buffer purchased from Shang Hai Gefan Biotechnology Co., Ltd. was used to remove red blood cells. Lymphocyte separation medium (No. 150624) was produced by Shanghai Yuanye Biology Inc. Formalin, dimethylbenzene, absolute alcohol, hematoxylin, hydrochloric acid, eosin staining solution, glycerol, ethanol and acetone were produced by Sinopharm Chemical Reagent Co., Ltd. Aprotinin (CAS 9087-70-1) was purchased from Solarbio Science & Technology Co., Ltd. FITC-conjugated rabbit anti-goat IgG was purchased from Beijing CW Biological Technology Co., Ltd. (CW0198, 1:25~100). Goat anti-chicken IgA antibody was purchased from Abcam Company (USA, ab120611, 1:1000). Goat serum working fluid was acquired from Sigma. The IgA enzyme-linked immunosorbent assay kit was acquired from Shanghai Lengton Biotechnology Co., Ltd. The ND vaccine (LaSota strain, No. 150306) was purchased from Qingdao Yibang Bioengineering Co., Ltd. ND virus (F48E9 strain) supplied by the China Institute of Veterinary Drug Control and was used for the hemagglutination inhibition test. Chicken IL-2 and IFN-γ enzyme-linked immunosorbent assay (ELISA) kits were products from Shanghai Langdun Biotechology Inc.

### Isolation and purification of the polysaccharides

Crude MLPs (200 mg) were dissolved in 10 mL of distilled water, and the solution was filtered through a 0.45 μm membrane. The filtrate was passed through an anion exchange column (2.6 cm×50 cm) of DEAE-52 cellulose. The eluents were 0.1, 0.3, and 0.5 mol/L NaCl at a flow rate of 1 mL/min. The total sugar content of the eluate was determined by the phenol-sulfuric acid method [16]. The fractions eluted with different concentrations of NaCl were pooled, desalted and further purified on a Sephadex G-100 column eluted with distilled water at 0.5 mL/min. The major polysaccharide fractions were pooled, concentrated and lyophilized. This process afforded the purified polysaccharides, which were further characterized.

### Analysis of the monosaccharide compositions

The monosaccharide compositions were determined by high-performance liquid chromatography (HPLC) after precolumn derivatization. Purified polysaccharide powder (20 mg) was dissolved in trifluoroacetic acid at 2 mol/L and hydrolyzed at 120°C for 6 hours in a sealed tube. After hydrolysis, the excess acid was removed by codistillation with methanol three times to yield dry hydrolysate. Then, the hydrolysate was derivatized with PMP and analyzed by HPLC according to a previously reported method [17]. HPLC analysis was performed on an LC20A HPLC system (Shimadzu, Japan) equipped with an SPD-20A ultraviolet detector and a C18 column (250 mm×4.6 mm, 5 μm, Shimadzu, Japan). The mobile phase was a mixture of 0.1 mol/L NaH_2_PO_4_-Na_2_HPO_4_ buffer (pH 6.7) and acetonitrile (83:17), and a flow rate of 1.0 mL/min was used. The wavelength of detection was 245 nm, and the column temperature was 30°C. The sugars were identified by comparison to reference monosaccharides (mannose, glucose, D-ribose, rhamnose, D-xylose, D-galactose, L-arabinose, and D-fructose). The molar ratios of the monosaccharides were calculated based on the standard curve of each monosaccharide.

### Determination of the molecular weight

The molecular weights of the MLP fractions were determined by high-performance gel permeation chromatography (HPGPC) using an Agilent 1200 HPLC system equipped with an evaporative light scattering detector and a TSK-gel 4000 PWXL column (7.8 mm×30 cm, TOSOH Corp., Japan). The column was eluted with double-distilled water at a flow rate of 0.6 mL/min. Standard dextrans (T10, T40, T70, T380, and T500) were used for the molecular weight determinations.

### Infrared spectroscopy

Fourier transform infrared spectra of the polysaccharides were acquired on a Bruker-Vector 22 spectrometer (Germany). The polysaccharides were mixed with KBr powder, ground and pressed into 1 mm pellets, and spectra were acquired in the frequency range of 4000–500 cm^-1^.

### UV spectrometry

A total of 1 mg of each purified polysaccharide was dissolved in 5 mL of distilled water and filtered through a 0.45 μm membrane. UV wavelength scans in the range of 190~300 nm were performed to determine whether the polysaccharides contained nucleic acid and protein components. Nucleic acids have characteristic absorption peaks at 260 nm, and proteins have characteristic absorption peaks at 280 nm.

### *In vitro* splenocyte proliferation assay

#### Culture of mouse spleen lymphocytes

Six-week-old ICR mice were euthanized with CO_2_. After removing the surrounding connective tissue, spleens were transferred to a 200-mesh cell sieve wetted with PBS. Spleen cells were washed with PBS to form a single cell suspension, which was centrifuged at 1500 rpm for 10 min, and the supernatant was discarded. An equal volume of erythrocyte lysis solution was added to the spleen cell suspension. The mixture was centrifuged at 1500 rpm for 10 min, and the supernatant was discarded again. The precipitate was collected and washed twice with PBS, and then the cell density was adjusted to 4.0×10^6^ cells/mL with RPMI-1640. This solution was used as the mouse spleen lymphocyte suspension.

#### Experiments on the effects of MLP on T lymphocyte proliferation stimulated with PHA in mice

The above spleen lymphocyte suspension was transferred to a 96-well plate at 80 μL/well, and 20 μL of PHA was added to half of the wells (final concentration of 10 μg/mL), whereas the other half remained without PHA. Then, 100 μL of MLP-1 and MLP-2 solutions were added to each well (final concentrations of 15.625 μg/mL, 31.25 μg/mL, 62.5 μg/mL, 125 μg/mL, and 250 μg/mL), and each concentration was tested in 4 wells. A PHA control, a cell control and a blank well were used. All cell plates were cultured in a carbon dioxide incubator at 37°C with 5% CO_2_ for 44 hours. MTT (30 μL) was added to each well, and the plates were incubated for an additional 4 hours. The supernatant was removed, and 100 μL of DMSO was added to each well. The cell plates were exposed to light for 5 min to completely dissolve the precipitate. The *A*_*570*_ values were tested as an indicator of T lymphocyte proliferation.

#### Experiments on the effects of MLP on B lymphocyte proliferation stimulated with LPS in mice

The above spleen lymphocyte suspension was transferred to a 96-well plate at 80 μL/well, and 20 μL of LPS (final concentration of 10 μg/mL) was added to half of the wells, whereas half remained without LPS. Then, 100 μL of MLP-1 and MLP-2 solutions were added to each well (final concentrations of 15.625 μg/mL, 31.25 μg/mL, 62.5 μg/mL, 125 μg/mL, and 250 μg/mL), and each concentration was tested in 4 wells. An LPS control, a cell control and a blank well were prepared. All cell plates were cultured in a carbon dioxide incubator at 37°C with 5% CO_2_ for 44 hours. MTT (30 μL) was added to each well, and the plates were incubated for an additional 4 hours. The supernatant was removed, and 100 μL of DMSO was added to each well. The cell plates were exposed to light for 5 min to dissolve the precipitate. The *A*_*570*_ values were tested as an indicator of B lymphocyte proliferation.

### Acute oral toxicity studies

#### Animals

Ninety healthy ICR mice (aged 4 weeks, 1:1 male:female ratio, weight 20±2 g) were obtained from Yangzhou University Comparative Medicine Center (Approval No. SCXK(su) 2012–0004, Yangzhou, China). The mice were housed under controlled temperature (24±2)°C and humidity (60±20) % conditions and allowed free access to food and water. Before the experiments, the mice were adapted to the environment for seven days. The mice were fasted for 12 hours before the test but were still allowed to drink water freely.

#### Pre-experiment

Fifty mice were randomly divided into five groups (10 in each group, 5 per sex). Mice in the normal control (NC) group were administered distilled water by oral gavage, and mice in the treated groups were administered MLP-2 at doses of 4.0, 2.0, 1.0, 0.5 g/kg body weight once in 24 hours. The mice were observed for seven successive days, including their diet, actions and mortality. The results showed that all mice were healthy and that there was no mortality. Therefore, LD_50_ could not be determined. According to the technical specification for acute toxicity tests (Measures for the registration and administration of new veterinary drugs, China, 2013), the maximum tolerated dose (MLD) of MLP-2 was tested in mice.

#### MLD test

Forty mice were randomly divided into two groups (20 in each group, 10 per sex). Mice in the normal control (NC) group were administered distilled water by oral gavage. Mice in the treated groups were administered MLP-2 at a dose of 4.0 g/kg body weight three times in 24 hours. At 6 hours after the initiation of administration, general symptoms of toxicity and mortality were observed and recorded once a day in the following two weeks. The changes in body weight before administration and the fourteenth day after administration were also recorded. On the 14th day after administration, all mice were anesthetized, and approximately 0.75 mL of blood was collected by extirpating the eyeballs for blood and biochemical tests. Hematologic indicators included white blood cell (WBC), red blood cell (RBC), platelet count (PLT), hemoglobin (HB), lymphocyte (LY), hematocrit (HCT), and plateletcrit (PCT). Serum biochemical indicators included alanine transaminase (ALT), alanine transaminase (AST), blood urea nitrogen (BUN) and alkaline phosphatase (ALP). Then, the mice were sacrificed, and a complete necropsy was conducted. The presence of lesions and any abnormality of the internal organs, such as the heart, liver, spleen, lung, kidney, testis, and uterus, were observed.

### Design of *in vivo* experiments

#### Animals

One-day-old white egg chickens (male), purchased from Jiangsu Institute of Poultry Sciences, were raised in an air-conditioned room at 37°C. At the beginning of the experiment, the light was continuously on, but light exposure was gradually reduced to 12 hours per day until the animals were in total darkness at night. The chickens were fed standard feed purchased from Taizhou Zhengda Feed Co., Ltd. and had free access to drinking water.

#### Serum IL-2 and IFN-γ assay

On days 7 (D_7_), 14 (D_14_), 21 (D_21_) and 28 (D_28_) after the first vaccination, blood samples were collected randomly from four chickens of each group for determining the serum contents of IL-2 and IFN-γ using ELISA kits.

#### Experiments on MLP-2 enhancement of ND vaccine immune response

One hundred and eighty 14-day-old chickens of similar weight (approximately 80~90 g) were randomly divided into 9 groups and immunized with ND vaccine by the nose- and eye-dropping method except for those in the blank control (BC) group, which was not immunized. Repeated immunization was carried out at the age of 28 days. Between the first and second immunizations, the MLP-2 groups were orally administered 0.5 mg, 1 mg, 2 mg, 4 mg, 8 mg and 12 mg of MLP-2, and the chickens in the APS group were orally administered 4 mg of APS for seven consecutive days. Chickens in the vaccination control (VC) group and BC group were not administered any polysaccharides. On days 7, 14, 21 and 28 after the first vaccination, blood samples were taken from ten chickens in each group to determine the serum HI antibody titer. On day 28 after the first vaccination, the average weight of the chickens in each group was determined using an electronic scale.

### Effects of MLP-2 on mucosal immunity in chickens

#### Animal experiments and experimental design

One hundred and eighty 14-day-old chickens were randomly divided into six groups. All chickens except for those in the blank control (BC) group were immunized with ND vaccine via the nose- and eye-dropping method, and the immunization was repeated at 28 days old. The chickens in the three purified MLP-2 groups were given MLP-2 at doses of 8 mg (MLP-2_H_), 4 mg (MLP-2_M_) and 2 mg (MLP-2_L_) at the same times as they were immunized. The APS group was orally administered APS at a dose of 4 mg. All drugs were administered once a day for seven consecutive days, and the VC and BC groups received nothing.

#### Determination of sIgA in jejunal and tracheal wash fluids

On days 7, 14, 21 and 28 after the first vaccination, six chickens were randomly selected from each group. The chickens were euthanized with CO_2_, and 5 cm sections of the jejunum and trachea were isolated from each chicken. The two ends of the intestinal canal and trachea were clipped with hemostatic forceps. PBS (0.5 mL, pH 7.4) containing aprotinin was injected into the samples. The washing liquor was fully extruded and mixed three times, collected in an Eppendorf tube, and centrifuged at 8000 rpm and 4°C for 10 min. The supernatant was stored at -20°C, and secretory IgA (sIgA) was determined by ELISA.

#### IgA^+^ cells counts in cecal tonsils

On days 7, 21 and 35 after the first vaccination, six chickens were randomly selected from each group. The chickens were euthanized with CO_2_. For each chicken, a one-centimeter section of cecal tonsil was removed and quickly fixed in 4% neutral formaldehyde solution; the fluid was exchanged 3 times at 12-hour intervals. The cecal tonsil section was embedded in paraffin, cut into slices and immunohistochemically stained with peroxidase streptavidin labeling. After dewaxing, rehydration, and antigen repair, goat serum and mouse anti-chicken IgA antibodies were added to the slides, and they were incubated overnight at 4°C. After washing with PBS, the biotinylated rabbit anti-mouse IgA secondary antibody was added, and the samples were incubated at 37°C for one hour. After hematoxylin staining, xylene treatment to make the samples transparent, and neutral gum sealing, the tissue structure was observed under a microscope. Image-Pro Plus 6.0 was used to analyze the tissue images, and the number of IgA^+^ cells in the cecal tonsils and trachea was counted.

### Statistical analysis

All values in the present study are expressed as the mean±standard deviations, and differences between means were considered significant at *p*< 0.05. Duncan’s multiple range test was used to determine the differences among groups using SPSS 18.0 software for analysis.

## Results

### Purification and characterization of MLP

#### Isolation and purification

**Fig 1:**
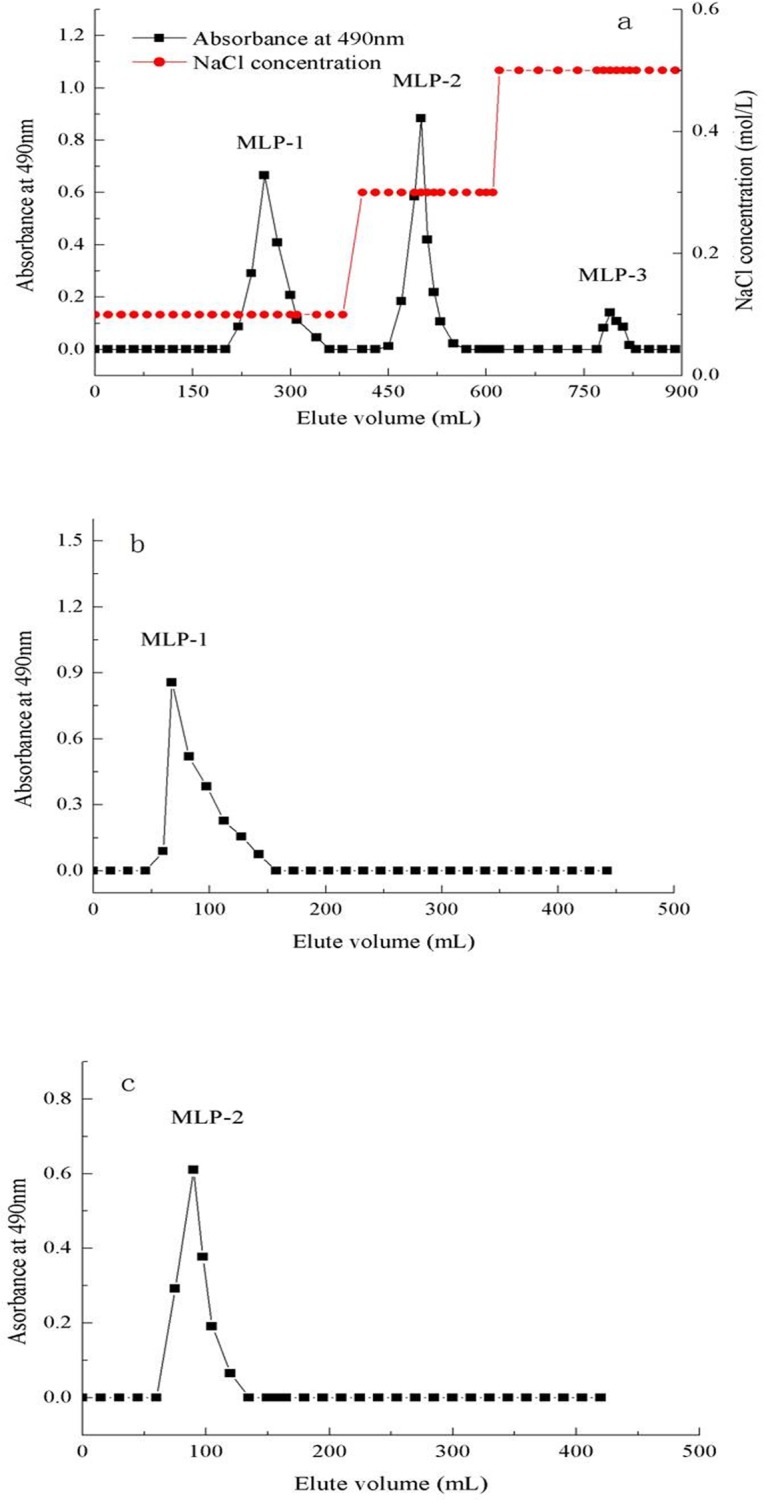
Elution chromatograms of the polysaccharides from mulberry leaves. (a) Elution curve of crude MLP from a DEAE-52 cellulose column; (b) elution curve of the MLP-1 fraction from a Sephadex G-100 column; and (c) elution curve of the MLP-2 fraction from a Sephadex G-100 column.

The crude polysaccharide was first fractionated using a DEAE-52 column. The main component of the polysaccharide fractions eluted with 0.1 and 0.3 mol/L NaCl was MLP (Fig 1A). Then, the fractions were further purified on a Sephadex G-100 column, and each fraction showed a single and symmetrical sharp peak (Fig 1B and Fig 1C). The major fractions were collected and lyophilized; thus, purified MLP-1 and MLP-2 were obtained.

#### Determination of the molecular weight

**Fig 2:**
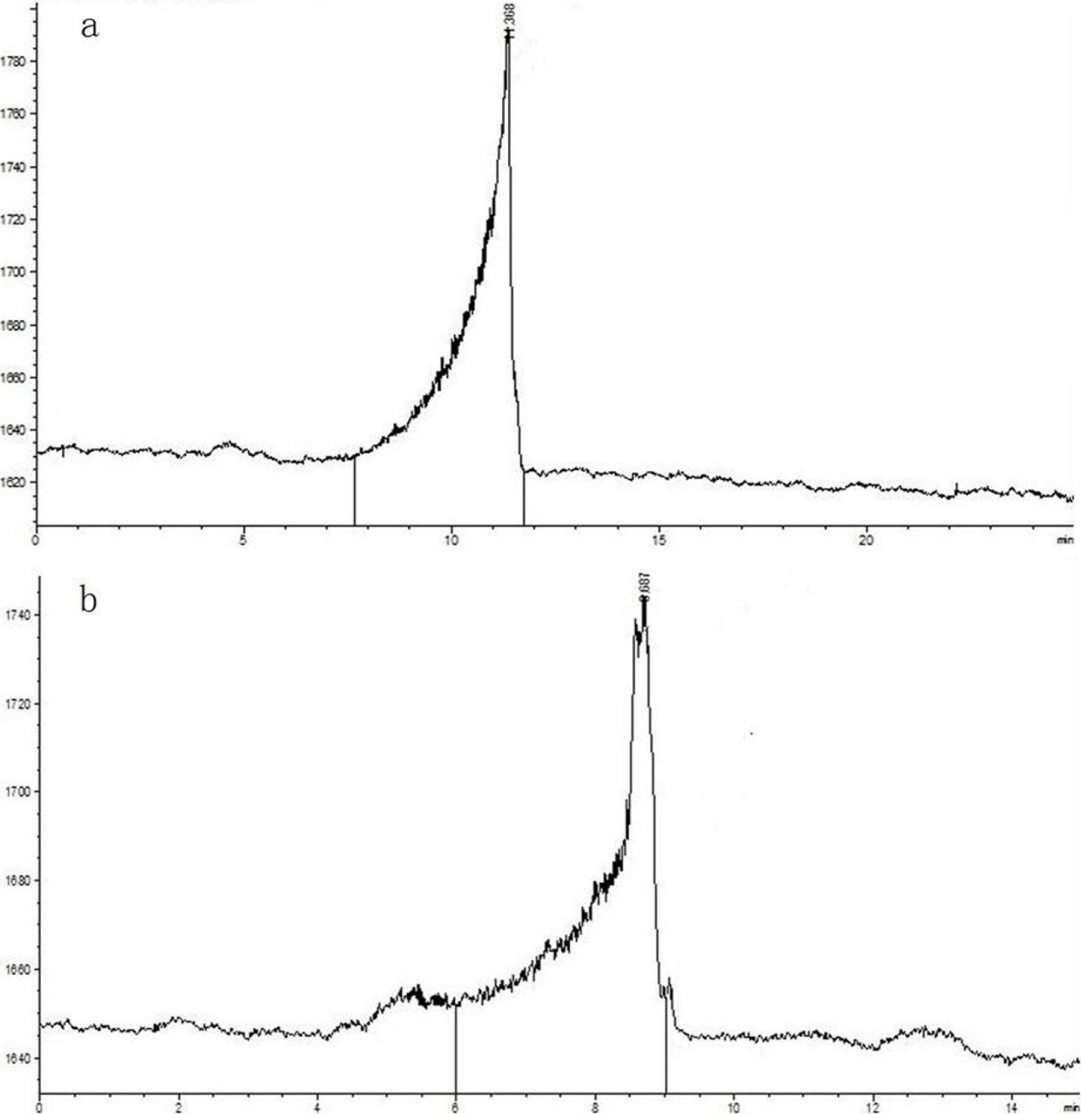
HPGPC chromatograms of MLP-1 (a) and MLP-2 (b).

As shown in Fig 2, a single and symmetrical peak was observed in the HPGPC chromatogram of each MLP. The retention times of MLP-1 (Fig 2A) and MLP-2 (Fig 2B) were 11.368 min and 8.687 min, respectively. The equation of the standard curve was logMw = -0.514x+7.809 (R^2^ = 0.992), where Mw is the molecular weight and x is the retention time.

#### Monosaccharide composition

**Fig 3:**
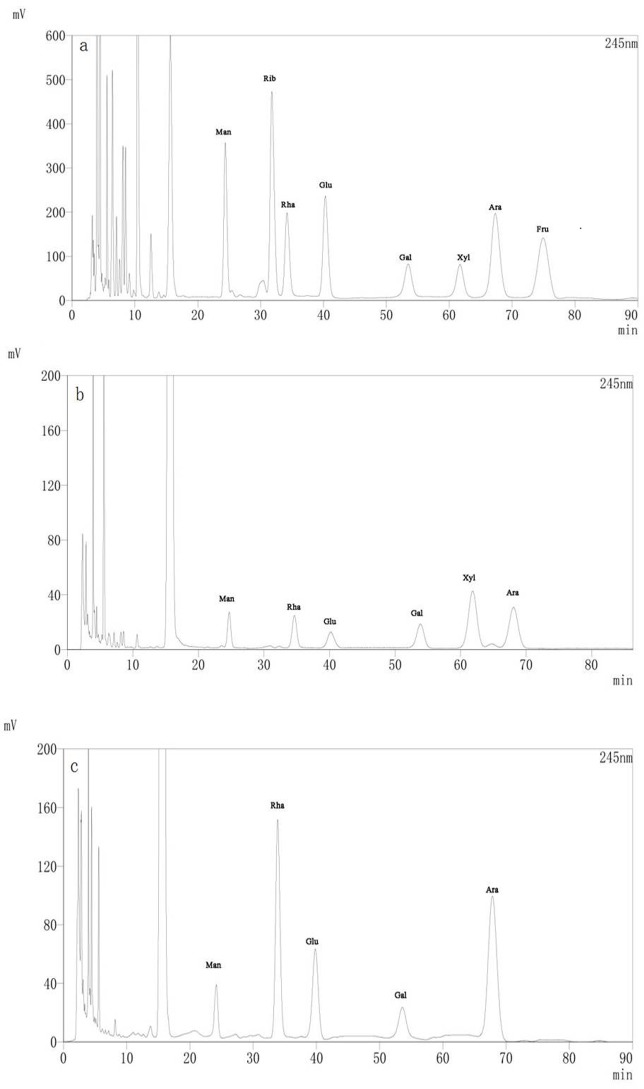
HPLC chromatograms of the PMP derivatives of the monosaccharide standards (a) and the hydrolysates of MLP-1 (b) and MLP-2 (c).

The monosaccharide compositions of MLP-1 and MLP-2 were measured by HPLC, as shown in Fig 3A. The retention time and standard curve for each monosaccharide were determined by HPLC analysis of standards of the individual components. The results indicated that MLP-1 consisted of mannose, rhamnose, glucose, galactose, xylose, and arabinose in a molar ratio of 0.71:1.00:2.76:1.13:3.70:2.81 (Fig 3B), while MLP-2 consisted of mannose, rhamnose, glucose, galactose, and arabinose in a molar ratio of 1.31:8.45:6.94:1.00:11.96 (Fig 3C).

#### Infrared spectrometry

**Fig 4:**
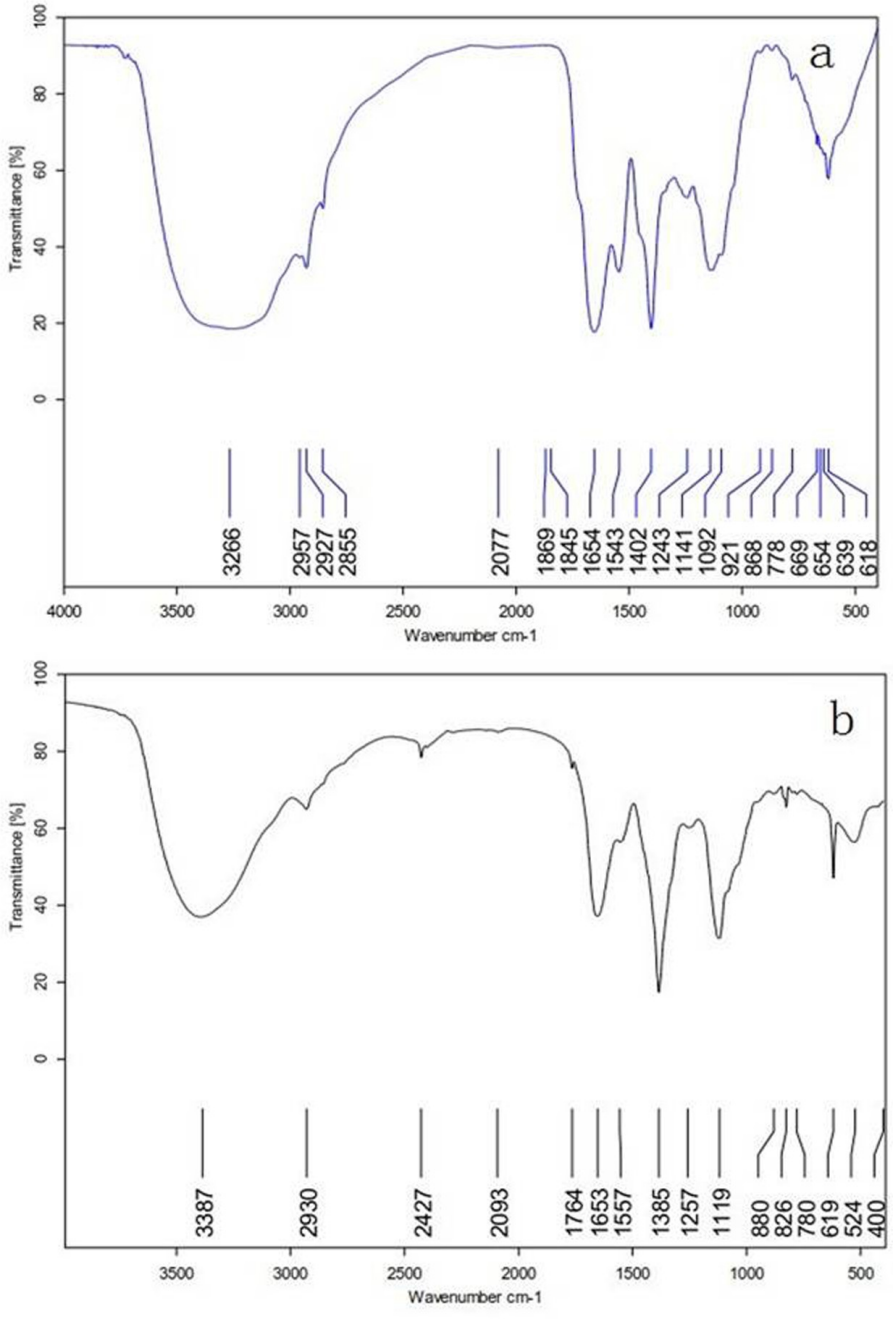
Infrared spectra of MLP-1 (a) and MLP-2 (b).

As shown in Fig 4, the broad and intense band at approximately 3350 cm^-1^ resulted from the stretching vibration of the hydroxyl groups. The signal at approximately 2930 cm^-1^ was assigned to the stretching vibration of the C-H bonds [18]. The signal at 1653 cm^-1^ was due to the bending vibrations of O-H, and the signals at approximately 1400 cm^-1^ and 1240 cm^-1^ were due to the bending vibrations of C-H and the stretching vibrations of C = O, respectively. The signal at approximately 1110 cm^-1^ was attributed to the stretching of the C-O-C linkages [19]. The absorption bands at 880 and 826 cm^-1^ of MLP-2 indicated the presence of β- and α-linked galactopyranosyl residues, respectively [20]. In addition, the signal in the spectrum of MLP-1 at 921 cm^−1^ was from β-type linkages [21].

#### UV spectrometry

**Fig 5:**
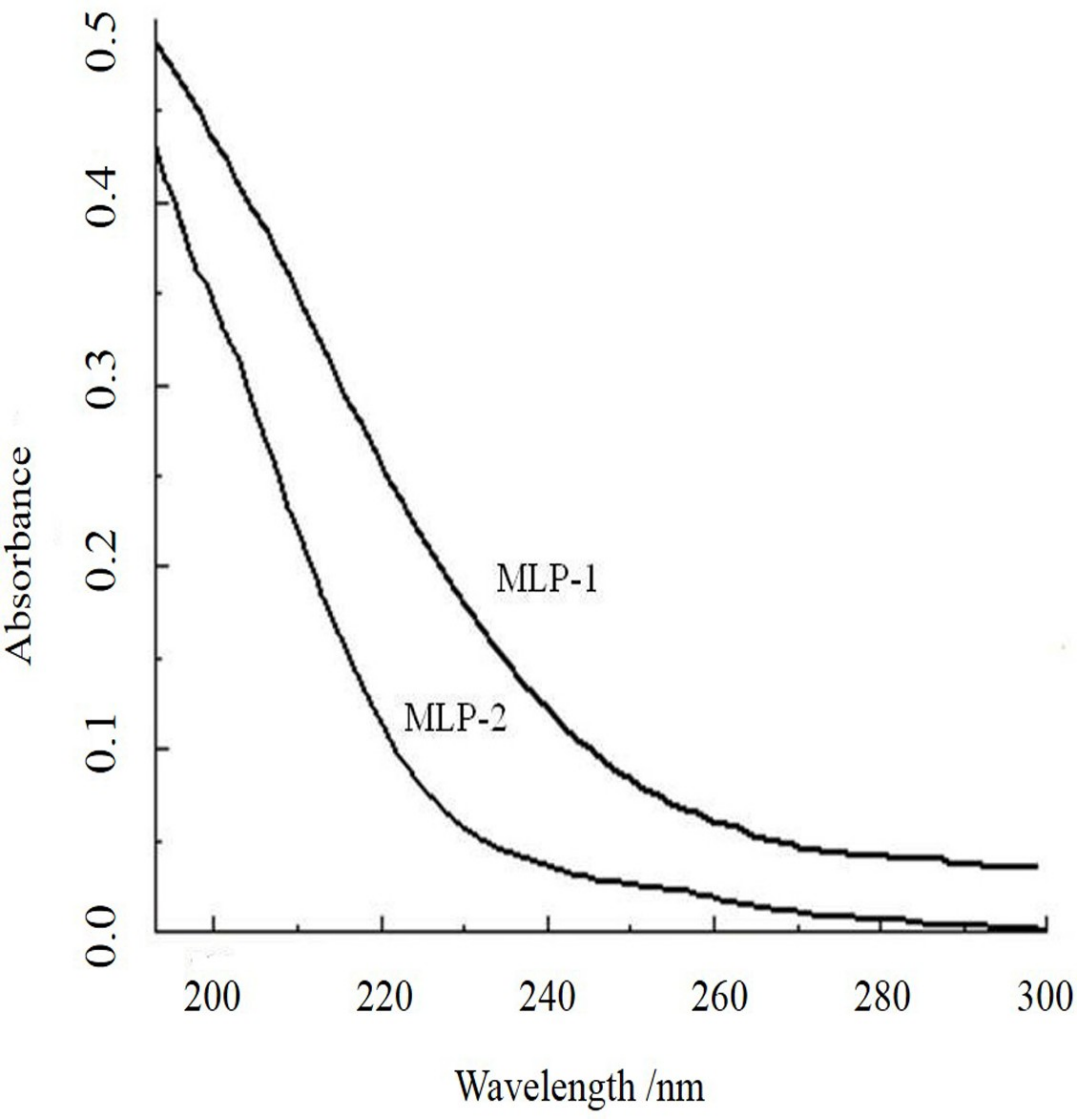
UV spectra of MLPs.

As shown in Fig 5, both MLP-1 and MLP-2 had no significant characteristic absorption peak at wavelengths of 260 nm and 280 nm. These results showed that the MLPs did not contain nucleic acid and protein component, indicating that purification by ion exchange and gel column chromatography could effectively remove impurities such as nucleic acids and proteins MLP.

### Effect of MLP on splenic lymphocyte proliferation

#### Effect of MLP on B lymphocytes stimulated with LPS

The *A*_*570*_ values of spleen B lymphocytes stimulated with LPS and treated with MLP are listed in Table 1. At all concentrations from 15.625–250 μg/mL, both MLP-1 and MLP-2 with LPS could stimulate B lymphocyte proliferation in the spleen. The *A*_*570*_ values of spleen B lymphocytes in the MLP-2-treated group were significantly higher than those in the MLP-1-treated, PHA control and cell control groups at all concentrations (*p*< 0.05). The results showed that MLP-2 was the most effective treatment for enhancing B lymphocyte proliferation stimulated by LPS.

**Table 1:** Effect of MLPs on B lymphocytes stimulated with LPS (A_570_).

Groups	Concentration (μg/mL)				
	15.625	31.25	62.5	125	250
MLP-1	0.441±0.025^b^	0.450±0.020^b^	0.466±0.020^b^	0.465±0.029^b^	0.426±0.029^b^
MLP-2	0.508±0.013^a^	0.508±0.019^a^	0.557±0.029^a^	0.563±0.022^a^	0.551±0.019^a^
LPS	0.352±0.025^c^	0.352±0.025^c^	0.352±0.025^c^	0.352±0.025^c^	0.352±0.025^c^
CC	0.267±0.015^d^	0.267±0.015^d^	0.267±0.015^d^	0.267±0.015^d^	0.267±0.015^d^

Data in the same column without the same superscript (a-d) differ significantly (*p*< 0.05).

#### Effect of MLP on T lymphocyte proliferation stimulated with PHA

The *A*_*570*_ values of spleen T lymphocytes stimulated with PHA after treatment with MLP are listed in Table 2. In the concentration range from 15.625 to 250 μg/mL, the *A*_*570*_ values of spleen T lymphocytes in the MLP-2-treated group were significantly higher than those in the MLP-1-treated, PHA control and cell control groups (*p*< 0.05). The results of the *in vitro* experiments demonstrated that MLP-2 was the most effective in improving T lymphocyte proliferation after stimulation with PHA.

**Table 2:** Effect of MLP on T lymphocyte proliferation stimulated with PHA (A_570_).

Groups	Concentration (μg/mL)				
	15.625	31.25	62.5	125	250
MLP-1	0.364±0.018^b^	0.383±0.027^b^	0.391±0.013^b^	0.375±0.019^b^	0.394±0.016^b^
MLP-2	0.409±0.015^a^	0.444±0.016^a^	0.448±0.018^a^	0.461±0.011^a^	0.460±0.014^a^
PHA	0.337±0.012^c^	0.337±0.012^c^	0.337±0.012^c^	0.337±0.012^c^	0.337±0.012^c^
CC	0.267±0.015^d^	0.267±0.015^d^	0.267±0.015^d^	0.267±0.015^d^	0.267±0.015^d^

Data in the same column without the same superscript (a-d) differ significantly (*p*< 0.05).

#### Acute toxicity assay

During the course of the experiment, no animals died, and no noticeable deviations in general behaviors or abnormalities of the internal organs were observed. The body weight change results are presented in Table 3. There were no significant differences in body weight changes between the NC and treated groups. The effects of MLP-2 on hematologic parameters in blood are shown in Table 4. No significant differences in hematologic indicators were found between the NC and MLP-2 treated groups. The effects of MLP-2 on biochemical indicators in serum are shown in Table 5. No significant differences in ALT, AST, BUN or ALP were found between the NC and MLP-2 treated groups.

**Table 3:** Effects of MLP-2 on body weight of mice (n = 10).

Group	Dose	0 days (g)	After 7 days (g)	After 14 days (g)
MLP-2 (female)	4 g/kg	20.14±1.25	24.58±1.67	28.16±1.97
BC (female)	-	20.10±0.87	24.88±0.96	26.66±1.02
MLP-2 (male)	4 g/kg	21.62±0.34	26.36±2.38	28.98±3.26
NC (male)	-	20.42±1.40	26.98±2.74	30.98±2.25

**Table 4:** Effects of MLP-2 on hematologic indicators in mice (n = 20).

Group	WBC (×10^9^/L)	RBC (×10^12^/L)	PLT (×10^9^/L)	HB (g/L)	LY (%)	HCT (L/L)	PCT (L/L)
MLP-2	4.86±0.54	8.67±0.52	811.07±52.34	135.40±4.56	68.18±9.98	39.05±1.09	0.21±0.02
NC	4.83±0.16	8.71±0.46	813.43±53.54	134.37±5.01	71.52±8.04	41.23±1.02	0.20±0.07

**Table 5:** Effects of MLP-2 on serum biochemical indicators in mice (n = 20).

Group	ALT (U/L)	AST (U/L)	BUN (mmol/L)	ALP (U/L)
MLP-2	44.34±4.26	106.21±7.32	6.21±0.23	101.34±3.78
NC	42.61±3.35	103.11±8.59	6.34±0.66	102.46±7.78

### *In vivo* tests

#### Changes in the ND antibody titer

The antibody titers of all groups are listed in Table 6. On days 7–28 after the first vaccination, the antibody titers in the MLP-2-treated (4 mg) group were significantly higher than those of the VC and BC groups (*p*< 0.05). On day 7, the titers in the MLP-2 (0.5 mg), MLP-2 (1 mg), MLP-2 (2 mg) and MLP-2 (4 mg) groups were slightly higher than those in the APS group but were significantly higher than those in the other groups (*p*< 0.05). From day 14 to day 28, the titers in the MLP (4 mg) group were higher than or significantly higher than those in the other groups. On day 21 and day 28, the titers in the MLP-2 (4 mg) group were significantly higher than those in the APS group (*p*< 0.05). Over the whole observation period, the titers in the VC group differed significantly from those in the BC group (*p*< 0.05).

**Table 6:** Changes in the antibody titer in the ND vaccination experiment (Log_2_) (n = 10).

Group	D_7_	D_14_	D_21_	D_28_
MLP-2 (0.5 mg)	4.70±0.213^a^	5.80±0.512^ab^	7.30±0.300^ab^	5.11±0.309^b^
MLP-2 (1 mg)	4.90±0.379^a^	5.20±0.593^b^	7.00±0.365^ab^	5.09±0.277^b^
MLP-2 (2 mg)	4.60±0.427^a^	5.30±0.213^b^	6.90±0.277^ab^	5.10±0.379^b^
MLP-2 (4 mg)	4.70±0.260^a^	6.50±0.654^a^	7.67±0.500^a^	5.80±0.213^a^
MLP-2 (8 mg)	4.30±0.133^b^	4.70±0.423^bc^	6.60±0.306^b^	5.60±0.452^a^
MLP-2 (12 mg)	4.20±0.249^b^	4.50±0.866^c^	7.20±0.249^ab^	5.40±0.211^ab^
APS	4.50±0.269^ab^	5.60±0.636^ab^	6.67±0.367^b^	5.15±0.340^b^
VC	4.10±0.314^b^	5.00±0.367^bc^	6.30±0.260^b^	5.12±0.221^b^
BC	1.50±0.373^d^	0.60±0.267^d^	0.40±0.221^c^	1.10±0.277^c^

Data in the same column without the same superscript (a-d) differ significantly (*p*< 0.05).

#### The changes in the average body weight

On day 28 after the first vaccination, the average weights in the 0.5 mg, 1 mg, 2 mg, 4 mg, 8 mg and 12 mg MLP-2-treated groups and in the APS, VC and BC groups were (438.6±16.9) g, (421.3±21.2) g, (418.0±18.4) g, (459.6±35.1) g, (438.1±21.4) g, (438.5±9.9) g, (432.3±17.8) g, (415.8±7.2) g and (407.9±3.5) g, respectively. The average weight in the MLP-2 (4 mg) group was highest, and this weight was significantly higher than those in the MLP-2 (1 mg), MLP-2 (2 mg), VC and BC groups (*p*< 0.05).

#### Changes in IL-2 concentration

The changes in serum IL-2 concentration in each group are shown in Table 7. On day 21, the IL-2 content of each group reached a peak value and gradually decreased after 21 days. On days 14 to 28 after first immunization, the IL-2 content of the MLP-2_H_, MLP-2_M_ and APS groups was significantly higher than that of the MLP-2_L_, VC and BC groups (*p*< 0.05), though there was no significant difference among the MLP-2_H_, MLP-2_M_ and APS groups (*p*> 0.05).

**Table 7:** The IL-2 concentration in each group (ng/L).

Group	D_7_	D_14_	D_21_	D_28_
MLP-2_H_	0.752±0.064^a^	0.985±0.041^a^	1.281±0.062^a^	1.213±0.341^a^
MLP-2_M_	0.785±0.048^a^	1.012±0.067^a^	1.305±0.016^a^	1.279±0.124^a^
MLP-2_L_	0.764±0.037^a^	0.824±0.032^b^	0.972±0.041^b^	0.922±0.039^b^
APS	0.711±0.058^a^	0.953±0.023^a^	1.284±0.101^a^	1.271±0.026^a^
VC	0.745±0.073^a^	0.735±0.042^c^	0.871±0.112^bc^	0.825±0.013^b^
BC	0.715±0.078^a^	0.713±0.054^c^	0.707±0.049^c^	0.758±0.102^b^

Data in the same column without the same superscript (a-c) differ significantly (*p*< 0.05). H, high dose; M, medium dose; L, low dose.

#### Changes in IFN-γ concentration

As shown in Table 8, on day 7, the IFN-**γ** concentration of the MLP-2_H_ group was significantly higher than that of the MLP-2_M_, VC and BC groups (*p*< 0.05); there was no significant difference among MLP-2_H_, MLP-2_M_ and APS *(p*> 0.05). On day 14, the IFN-**γ** concentration of both MLP-2_H_ and MLP-2_M_ was significantly higher than that of the other groups (*p*< 0.05). On day 28, IFN-**γ** concentrations in the MLP and APS groups were significantly higher than those of the VC and BC groups, yet there was no significant difference among MLP-2_H_, MLP-2_M_ and APS (*p*> 0.05).

**Table 8:** The IFN-γ concentration in each group (ng/L).

Group	D_7_	D_14_	D_21_	D_28_
**MLP-2_H_**	**1.153±0.043^a^**	**1.211±0.034^a^**	**1.863±0.024^a^**	**1.573±0.098^a^**
**MLP-2_M_**	**1.187±0.051^a^**	**1.213±0.056^a^**	**1.875±0.032^a^**	**1.525±0.076^a^**
**MLP-2_L_**	**1.031±0.032^b^**	**0.971±0.011^b^**	**1.682±0.018^b^**	**1.278±0.081^b^**
**APS**	**1.164±0.047^a^**	**1.198±0.072^a^**	**1.881±0.054^a^**	**1.545±0.021^a^**
**VC**	**0.908±0.023^b^**	**0.902±0.036^b^**	**0.846±0.047^c^**	**0.858±0.033^c^**
**BC**	**0.772±0.076^c^**	**0.635±0.018^c^**	**0.749±0.023^c^**	**0.764±0.079^c^**

Data in the same column without the same superscript (a-c) differ significantly (*p*< 0.05). H, high dose; M, medium dose; L, low dose.

### Effects of MLP-2 on mucosal immune function

#### Changes in the sIgA levels in the jejunal intestinal wash fluid

**Fig 6:**
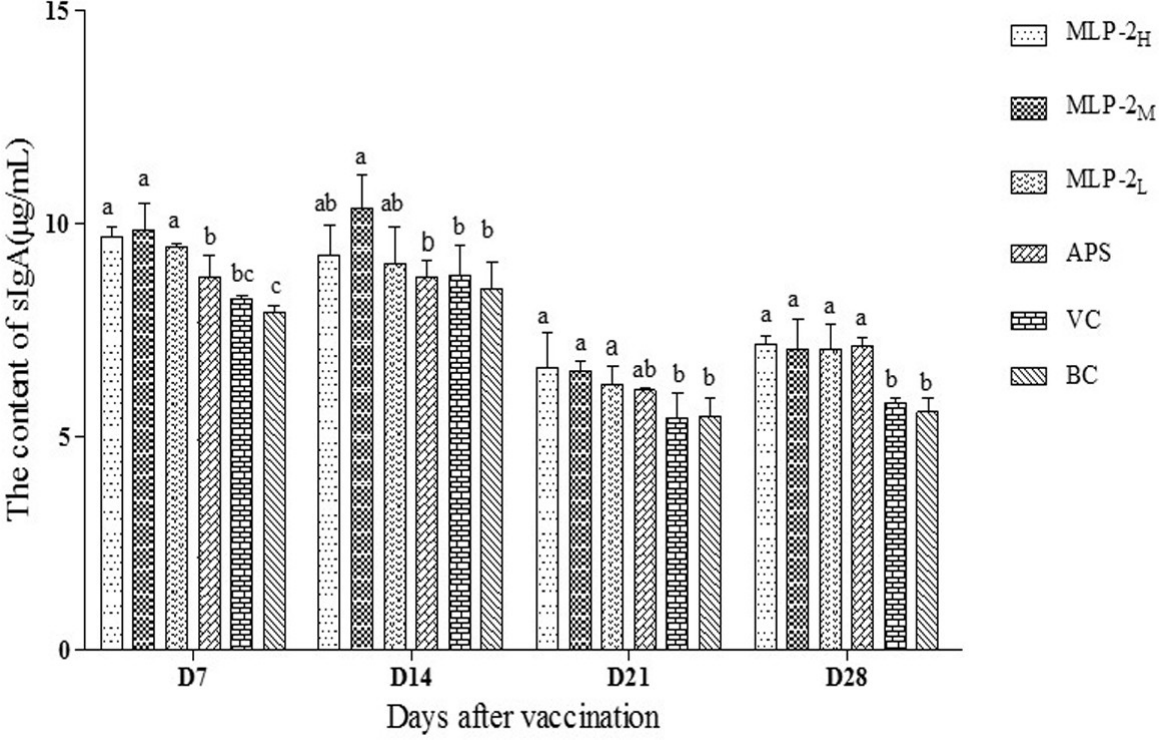
The sIgA levels in the jejunal wash fluids from each group (μg/mL) (n = 6). Bars without the same superscript (a-c) differ significantly (*p*< 0.05).

The sIgA levels in the jejunal wash fluids from different groups are shown in Fig 6. On day 7, the sIgA levels in the MLP-2_H_, MLP-2_M_ and MLP-2_L_ groups were significantly higher than those in the APS, VC and BC groups (*p*<0.05). On day 14, the sIgA levels in the MLP-2_M_ group were significantly higher than those in the other groups except for the MLP-2_H_ and MLP-2_M_ groups (*p*< 0.05). On day 21, the sIgA levels in the MLP-2_H_, MLP-2_M_ and MLP-2_L_ groups were significantly higher than those in the other groups except for the APS group (*p*< 0.05). On day 28, the levels of sIgA in all drug-treated groups were significantly higher than those in the VC and BC groups (*p*< 0.05), but there were no significant differences among the MLP-2 groups (*p*> 0.05).

#### Changes in the sIgA levels in the tracheal wash fluids

**Fig 7:**
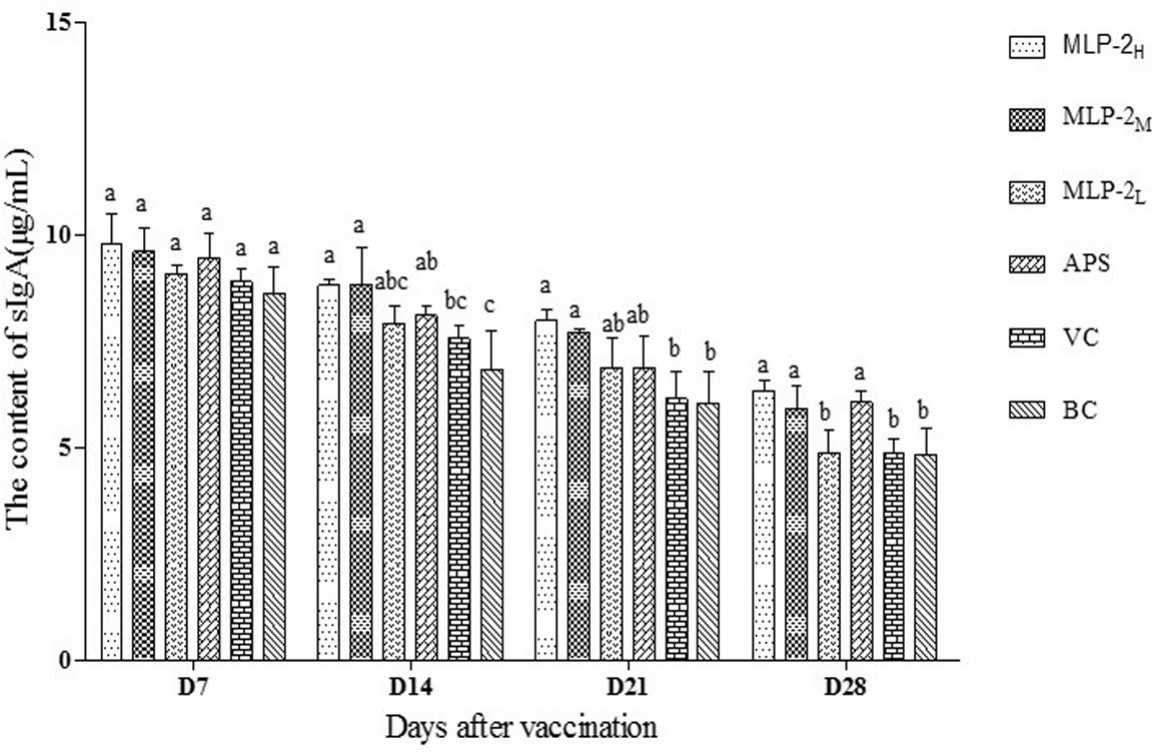
The sIgA levels in the trachea washing liquids from each group (μg/mL) (n = 6). Bars without the same superscript (a-c) differ significantly (*p*< 0.05).

The sIgA levels in the tracheal wash fluids from each group are shown in Fig 7. On day 7, the sIgA levels in the tracheal wash fluids in each MLP-2 group were higher than those in the VC group and the BC group, but the differences were not significant (*p*>0.05). On day 14 and day 21, the sIgA levels in the MLP-2_H_ and MLP-2_M_ groups were somewhat higher than those in the other groups and were significantly higher than those in the VC and BC groups (*p*< 0.05). On day 28, the sIgA levels in the MLP-2_H_, MLP-2_M_ and APS groups were significantly higher than those in the other groups (*p*< 0.05).

#### Changes in the IgA^+^ cell number in the cecal tonsil

**Fig 8:**
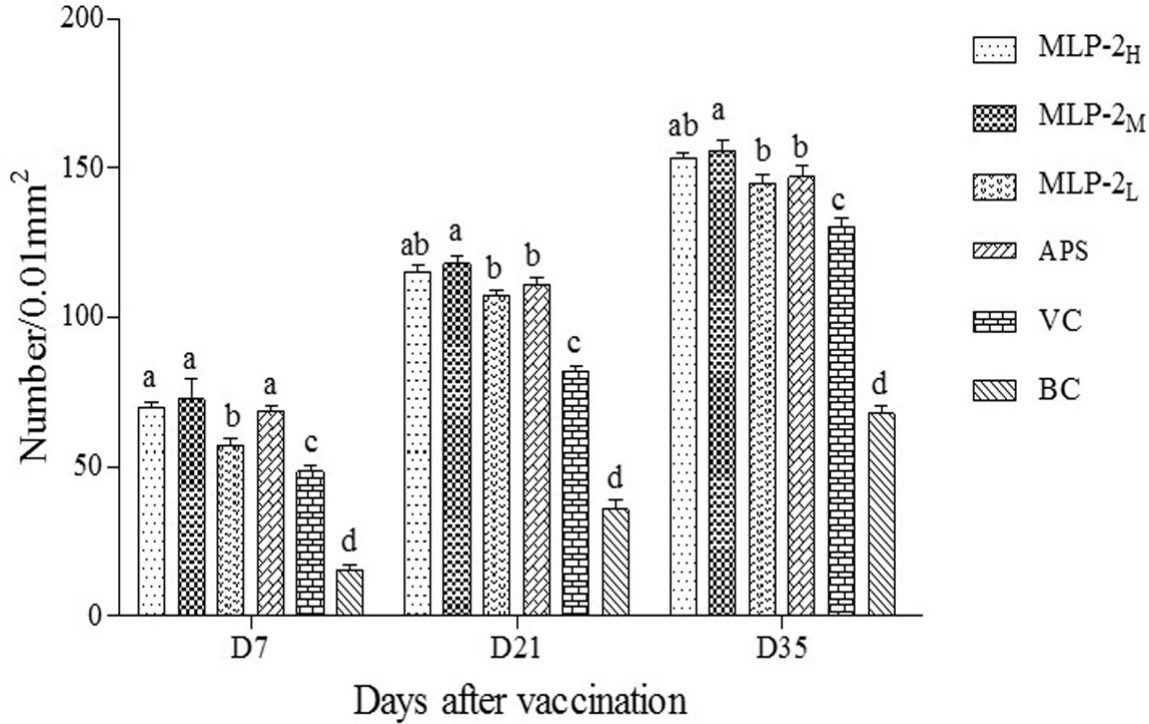
IgA^+^ cell numbers in the cecal tonsils of each group (n = 6). Bars without the same superscript (a-d) differ significantly (*p*< 0.05).

The numbers of IgA^**+**^ cells in the cecal tonsils of each group are shown in Fig 8. Across the three time points following the first vaccination, the number of IgA^**+**^ cells in the cecal tonsils of each group increased gradually. On day 7, the IgA^+^ cell numbers in the MLP-2_H_, MLP-2_M_ and APS groups were significantly higher than those in the other groups (*p*< 0.05). On days 21 and 35, the IgA^**+**^ cell numbers in the MLP-2_M_ group were significantly larger than those in the other groups except for the MLP-2_H_ group (*p*< 0.05).

## Discussion

In this experiment, MLPs were isolated and purified to afford MLP-1 and MLP-2. Compositional analysis showed that MLP-1 and MLP-2 had similar monosaccharide constituents but differed significantly in monosaccharide contents, as MLP-1 contained xylose, while MLP-2 did not. In our study, the compositions of the MLPs were different from the previously reported values [22], and this discrepancy may be due to different growth environments [23]. Based on the regression equation, the average molecular weights of MLP-1 and MLP-2 were calculated as 9.31×10^4^ Da and 2.22×10^6^ Da with retention times of 11.368 min and 8.687 min, respectively. Absorption peaks characteristic of sugars appeared in the infrared spectra of both MLP-1 and MLP-2, and UV spectroscopy showed that the MLPs did not contain nucleic acid or protein components.

The effects of purified MLP-1 and MLP-2 on the *in vitro* proliferation of immune cells were studied. The spleen, with its hematopoietic, immune response and other functions, is an important lymphoid organ; T and B lymphocytes accumulate in the spleen, and they play key roles in cellular and humoral immunity [24]. T and B lymphocytes, with specific antigen receptors, can give specific immune responses after stimulation by an antigen and subsequent proliferation and differentiation. In this study, MLP-2 at all tested *in vitro* concentrations (15.625–250 μg/mL) could synergistically interact with LPS and PHA to promote spleen B and T lymphocyte proliferation, respectively, to a significantly greater degree than the MLP-1, LPS, PHA and cell control treatments. Among the MLPs, MLP-2 showed the best *in vitro* immunostimulatory activity.

On the basis of the *in vitro* experiments, MLP-2 was selected as the experimental drug, and its acute oral toxicity and immunomodulatory functions were evaluated *in vivo*. When mice were administered MLP-2 at a dose of 4.0 g/kg body weight three times in 24 h, there was no significant difference in body weight, hematologic parameters and serum biochemical indicators compared to those in the normal group, which showed that MLP-2 had no acute oral toxicity in mice. Cytokines are essential immune mediators in regulating the immune response to natural infection and vaccination. After vaccination, serum cytokine levels correlate with the activation of IL-2 and IFN-**γ** and can mediate cellular immunity [25]. Our experimental results showed that the serum IL-2 and IFN-**γ** concentrations in the MLP-2_H_ and MLP-2_M_ groups were significantly higher than those of the VC and BC groups at all time points, with an action similar to that of APS. These results suggest that these polysaccharides effectively promote the secretion of immunocytokines. B-cell-mediated humoral immunity, an important specific immune response, is one of the main factors in infectious disease resistance. Peripheral serum antibody titers are indicators of humoral immune responses in animals, and these responses play an important role in the prevention and treatment of infectious diseases [24]. In this study, the antibody titers in the VC group were greater than 4 log2 for 7 to 28 days after the first vaccination and significantly higher than those in the BC group (*p*<0.05). These results showed that the low-virulence vaccine for Newcastle disease (LaSota strain) had better immunogenicity. However, when chickens immunized with the ND vaccine were orally administered MLP-2, the ND antibody titer could be greatly increased. On day 7 to day 28, the ND antibody titers and average weight in the MLP-2 (4 mg) group were higher or significantly higher than those in the other groups. These results showed that when chickens were orally administered MLP-2 at a dose of 4 mg for seven consecutive days before and after being vaccinated with the ND vaccine, their serum ND antibody titers could be significantly increased, their humoral immune response was enhanced, and their growth rate was accelerated.

The gastrointestinal and tracheal mucosal surfaces are the first line of defense against pathogenic microbial invasion [26]. Secretory IgA (sIgA) is the major antibody subtype in mucosal secretions, and it protects the mucosal surface in a variety of ways [27]. sIgA can reduce the colonization of many microorganisms on the mucosal surface of the host and effectively neutralize viruses and toxins produced in the intestine [28, 29]. This study investigated the effects of MLP-2 on the secretion of sIgA in jejunal mucus. The results showed that at each time point after the first vaccination, the levels of sIgA in the jejunal wash fluids from the MLP-2_H,_ MLP-2_M,_ and MLP-2_L_ groups were significantly higher than those from the VC and BC groups except on day 14 and were significantly higher than those from the APS group on day 7. The levels of sIgA in the MLP-2_M_ group were slightly higher than those of the other MLP-2 groups, but there were no significant differences among the MLP groups. The results suggested that MLP-2 could promote the secretion of sIgA in intestinal mucosal immune cells and can more effectively protect the intestinal tract than MLP-1.

This study also investigated the effects of MLP-2 on the secretion of sIgA in tracheal mucus. The results showed that on day 7 after the first vaccination, the levels of sIgA in the trachea wash fluids from the MLP-2_H,_ MLP-2_M,_ and MLP-2_L_ groups were higher than those from the VC and BC groups. From day 14 to day 21, the sIgA levels in the wash fluids from the MLP-2_H_ and MLP-2_M_ groups were significantly higher than those from the VC and BC groups, slightly higher than those from the MLP-2_L_ and APS groups, and significantly higher than those from the MLP-2_L_ group. Moreover, on day 28, the levels in the wash fluids from the MLP-2_H_ and MLP-2_M_ groups were similar to those from the APS group. The above results indicated that MLP-2 could promote the secretion of sIgA in the mucosal immune cells of the trachea, which might protect the respiratory tract against microbial invasion.

The cecal tonsils, which is at the base of the cecum, is located in the joint of the ileum, cecum and rectum where lymphoid tissue is well developed. The cecal tonsil is the largest intestinal-associated lymphoid tissue in poultry [30]. IgA^+^ cells present in its mucosal lamina propria can produce and release immunoglobulin IgA, which plays a critical role in mucosal protection and is vital in local immunity against intestinal bacteria and other antigens [31]. In this study, the IgA^+^ cell count results in cecal tonsils showed that the number of IgA^+^ cells in cecal tonsils gradually increased and reached a maximum at 35 days. From day 7 to day 35, the numbers of IgA^+^ cells in the three MLP-2 groups were significantly higher than those in the VC group, which indicated that MLP-2 could promote the production of cecal IgA^+^ cells and thus promote the secretion of sIgA. From day 21 to day 35, the numbers of IgA^+^ cells in cecal tonsils in the MLP-2_M_ group were significantly higher than those in cecal tonsils in the MLP-2_L_ and APS groups. The results showed that oral administration of MLP-2 at a dose of 4 mg in chickens could strongly enhance the formation of IgA^+^ cells. The use of MLP-2 as an immune adjuvant could further promote intestinal mucosa-associated lymphocyte proliferation and improve intestinal mucosal immune function. Similar results have been reported previously. For instance, the IgA^+^ cell number in the jejunum and cecal tonsil of chickens could be significantly increased by the oral administration of an appropriate dose of epimedium polysaccharide-propolis flavone oral liquid [32]. Ginseng stems and leaves of saponins orally administered to chickens could stimulate the duodenum and jejunum to secrete more IgA^+^ cells than were observed in the nontreated group [33].

## Conclusions

In this paper, the structural characteristics and immune-enhancing properties of MLP were investigated. MLP-1 contained mannose, rhamnose, glucose, galactose, xylose, and arabinose, and its molecular weight was 9.31×10^4^ Da. MLP-2 consisted of mannose, rhamnose, glucose, galactose, and arabinose, and its molecular weight was 2.22×10^6^ Da. Infrared spectral analysis of the two polysaccharides showed typical polysaccharide absorption bands, and neither MLP contained nucleic acid or protein components.

For immunocompetence, MLP-2 was more effective than MLP-1 in stimulating spleen lymphocyte proliferation in mice *in vitro*. An acute toxicity assay verified that MLP-2 had no acute toxicity in mice. The simultaneous oral administration of MLP-2 and vaccination with ND vaccine to chicken could significantly increase serum ND antibody titers, enhance the immune effect of the vaccine, and cause significant weight gain. MLP-2 could significantly promote the secretion of IL-2, IFN-γ, and sIgA in the jejunum and trachea, promote the production of IgA^+^ cells in cecal tonsils, and enhance the intestinal and tracheal mucosal immune function. Moreover, the effects of MLP-2 were stronger than those of APS, which suggested that MLP-2 could be developed as an oral immunopotentiator.

In recent years, some drug carrier systems, such as lipidosome and nanoparticle drug carriers, can improve precise drug targeting and release at the corresponding sites, which can enhance the therapeutic effect of the drug and lead to a lower dose requirement and a reduction in side effects [34, 35]. Future studies should focus on effective delivery of MLPs to make their *in vivo* application possible.
